# Dynamics of soil exploration by fine roots down to a depth of 10 m throughout the entire rotation in *Eucalyptus grandis* plantations

**DOI:** 10.3389/fpls.2013.00243

**Published:** 2013-07-09

**Authors:** Jean-Paul Laclau, Eder A. da Silva, George Rodrigues Lambais, Martial Bernoux, Guerric le Maire, José L. Stape, Jean-Pierre Bouillet, José L. de Moraes Gonçalves, Christophe Jourdan, Yann Nouvellon

**Affiliations:** ^1^CIRAD, UMR Eco&SolsMontpellier, France; ^2^Forest Science Department, UNESPBotucatu, Brazil; ^3^Forest Science Department, USP, ESALQPiracicaba, Brazil; ^4^Forest Science Department, UEMS, Universidade Estadual de Mato Grosso do SulCassilândia, Brazil; ^5^CENA, USPPiracicaba, Brazil; ^6^IRD, UMR Eco&SolsMontpellier, France; ^7^Department of Forestry and Environmental Resources, North Carolina State UniversityRaleigh, NC, USA; ^8^Atmospheric Sciences Department, USP, IAGSão Paulo, Brazil

**Keywords:** root front, root growth, root density, *Eucalyptus*, forest, oxisol, tropical tree, Brazil

## Abstract

Although highly weathered soils cover considerable areas in tropical regions, little is known about exploration by roots in deep soil layers. Intensively managed *Eucalyptus* plantations are simple forest ecosystems that can provide an insight into the belowground growth strategy of fast-growing tropical trees. Fast exploration of deep soil layers by eucalypt fine roots may contribute to achieving a gross primary production that is among the highest in the world for forests. Soil exploration by fine roots down to a depth of 10 m was studied throughout the complete cycle in *Eucalyptus grandis* plantations managed in short rotation. Intersects of fine roots, less than 1 mm in diameter, and medium-sized roots, 1–3 mm in diameter, were counted on trench walls in a chronosequence of 1-, 2-, 3.5-, and 6-year-old plantations on a sandy soil, as well as in an adjacent 6-year-old stand growing in a clayey soil. Two soil profiles were studied down to a depth of 10 m in each stand (down to 6 m at ages 1 and 2 years) and 4 soil profiles down to 1.5–3.0 m deep. The root intersects were counted on 224 m^2^ of trench walls in 15 pits. Monitoring the soil water content showed that, after clear-cutting, almost all the available water stored down to a depth of 7 m was taken up by tree roots within 1.1 year of planting. The soil space was explored intensively by fine roots down to a depth of 3 m from 1 year after planting, with an increase in anisotropy in the upper layers throughout the rotation. About 60% of fine root intersects were found at a depth of more than 1 m, irrespective of stand age. The root distribution was isotropic in deep soil layers and kriged maps showed fine root clumping. A considerable volume of soil was explored by fine roots in eucalypt plantations on deep tropical soils, which might prevent water and nutrient losses by deep drainage after canopy closure and contribute to maximizing resource uses.

## Introduction

Rooting depth is an important functional trait in terrestrial ecosystems. Meta-analyses have shown that the rooting depth for trees tends to be greater than for shrubs and grasses and that the maximum rooting depth in forest ecosystems is greater in equatorial regions than in boreal regions (Jackson et al., 1997; Schenk and Jackson, 2002a). Deep-rooted trees can have a strong influence on ecosystem services in tropical regions, both locally and globally. At a local scale, stream flows can be reduced after afforestation in grasslands and deep-rooted trees are important drivers of water cycling in dry ecosystems that can have a significant effect on landscape hydrology (Jackson et al., 2005; Bleby et al., 2010; Dye, 2012; Brown et al., 2013). At a global scale, modeling studies have shown that the current Amazonian climate is dependent on considerable amounts of water being extracted by trees from very deep soil layers and transpired back into the atmosphere during dry periods (Kleidon and Heimann, 2000; Saleska et al., 2007). A rainfall manipulation experiment showed that total carbon (C) stocks were strongly influenced by the availability of water in Amazonian forests (Brando et al., 2008) and the capacity of trees to take up water from deep soil layers during droughts (Bruno et al., 2006) can, therefore, influence C sequestration in rainforests. Although the major role of deep roots on C and water cycling has been described for several decades in tropical forest ecosystems (Nepstad et al., 1994), there are still few studies dealing with fine root development at depths greater than 5 m (Schenk and Jackson, 2002a,b, 2005; Christina et al., 2011).

*Eucalyptus* plantations cover about 20 million hectares and are expanding in tropical regions (Booth, 2013). Although considerable areas are concerned, there is still little information on the consequences of the afforestation of grasslands with *Eucalyptus* plantations on the storage of water, carbon, and nutrients in deep soil layers. The gross primary production (GPP) of commercial *Eucalyptus* plantations in Brazil is more than 3500 g C m^−2^ yr^−1^ (Ryan et al., 2010; Cabral et al., 2011; Nouvellon et al., 2012), among the highest in the world for forests (Luyssaert et al., 2007). This simple agro-ecosystem (with only 1 plant species growing in highly weathered soils without root growth barriers) provides an opportunity to investigate the belowground growth strategy of fast-growing trees in tropical regions. Most of the current information on tropical forests comes from indirect estimates of root activity from soil moisture monitoring (Calder et al., 1997; Robinson et al., 2006; Mendham et al., 2011) or tracer uptake (Lehmann, 2003; McCulley et al., 2004; da Silva et al., 2011). Spatial patterns of soil water depletion by *Eucalyptus* trees in Australian agroforests showed that *Eucalyptus* roots can take up water from the top soil up to 20 m from the tree belts and down to at least 8–10 m within 7 years after planting (Robinson et al., 2006). A recent study showed water uptake at a depth of 10 m 3.5 years after planting *Eucalyptus grandis* W. Hill ex Maiden trees in Brazil and a synchrony in vertical growth aboveground and belowground in very deep soils (Christina et al., 2011). Maps of fine root intersects counted in grids on vertical trench walls have been used to study the spatial distribution of roots in forest ecosystems (e.g., Laclau et al., 2001; Sudmeyer et al., 2004; Schmid and Kazda, 2005). This approach showed a tendency toward homogeneous soil exploration down to a depth of 3 m at 1 and 2 years after afforestation of a savanna with *Eucalyptus* trees in the Congo, followed by a concentration of fine roots in the upper soil layers at the end of the rotation period (Bouillet et al., 2002). However, fine roots were not observed beyond a depth of 3 m.

Our study aimed to gain an insight into the soil exploration strategy throughout the growth of *Eucalyptus* trees that enabled them to achieve the highest GPP in the world for forests. The study was based on the hypotheses that: (i) the root front velocity was at the uppermost range reported for tree species, as observed for eucalypt height growth, and (ii) most of the soil volume was explored by fine roots in the upper 3 meters from 1 year after planting onwards, which might explain the very low losses of mobile ions applied with fertilizers in these plantations (Laclau et al., 2010; Silva et al., 2013).

## Materials and methods

### Study site

The study was carried out in *E. grandis* plantations established at Itatinga, State of São Paulo (23°02′S, 48°38′W). The mean annual rainfall over the 15 years prior to this study was 1360 mm and the mean annual temperature was 20°C, with a seasonal cold period from June to September. The elevation was 850 m with a gently undulating topography typical of the São Paulo Western Plateau.

A chronosequence of *E. grandis* plantations covering an entire rotation cycle for pulpwood production (6 years) was studied on sandy soil (Table 1). The soils were deep Ferralsols (>10 m), developed on Cretaceous sandstone, with a clay content ranging from about 15% in the A_1_ horizon to 20–25% in deep soil layers. The mineralogy was dominated by quartz, kaolinite, and oxyhydroxides, with acidic soil layers containing very small amounts of available nutrients (see Campoe et al., 2012, for soil analyses). After harvesting a 10-ha *Eucalyptus* plot located on a hill top (slope <3%), plots (about 0.25 ha each) were planted every year with the same silvicultural practices, representative of commercial plantations.

**Table 1:** **Main characteristics of the stands sampled on sandy soil (chronosequence) and clayey soil**.

**Stand age (months)**	**Planting date**	**Soil type**	**# Soil profiles; (maximum depth)**	**Mean height (m)**	**LAI (m^2^ m^−2^)**
12	July 2006	Sandy soil	6 (1.5); 2 (6.0)	4.4	2.8
22	July 2005	Sandy soil	6 (1.5); 2 (6.0)	10.2	4.8
42	April 2004	Sandy soil	6 (3.0); 2 (10.0)	17.8	3.2
68	December 2002	Sandy soil	6 (3.0); 2 (10.0)	23.6	2.2
72	December 2002	Clayey soil	6 (3.0); 2 (10.0)	25.8	3.0

*The numbers of soil profiles and the maximum depth studied in each stand are indicated, as well as mean stand height and leaf area index (LAI)*.

*A complete description of the sandy soil (20% clay content) and the clayey soil (40% clay content) is given in Campoe et al. (2012)*.

Fine roots were studied within a radius of 300 m in 1-, 2-, and 3.5-year-old stands planted with *E. grandis* seedlings selected by the Suzano forest company. The youngest stand in the chronosequence was planted 1 year after the previous stand had been harvested. The area was kept free of other plants by successive glyphosate applications during the period between harvesting and planting. Management practices in Brazilian *Eucalyptus* plantations commonly use herbicide the first two years after planting to support tree growth through an efficient weed control (Gonçalves et al., 2008). Soil sampling in an adjacent unplanted area showed that the period of 2 years between clear cutting the previous stand and root sampling was sufficient to distinguish, without any doubt, between the living roots of the current stand and the dead roots of the previous stand. The roots from the previous rotation in the youngest stand of our chronosequence were already decomposed or in an advanced stage of decomposition, whatever the soil layer. All seedlings received standard commercial plantation fertilization, which was non-limiting for tree growth in this soil (120 kg N ha^−1^, 33 kg P ha^−1^, 100 kg K ha^−1^, 2 t ha^−1^ of dolomitic lime and micronutrients). Fertilizer was only applied on planting, except KCl and (NH_4_)_2_SO_4_ fertilizer, a quarter of the total amount being applied on planting with further applications at 6, 12, and 18 months of age. Experiments conducted over an entire rotation in Brazil showed that large amounts of fertilizers applied before canopy closure (as in our study) meet the nutritional demand of *Eucalyptus* trees up to the harvest age (Stape et al., 2010). The oldest stand of the chronosequence was sampled 6 years after planting. This stand was located 13 km away on the same type of soil and in a similar topographic position. Seedlings came from the same source with a similar planting strategy, except that the spacing was 1.6 × 3.8 m as opposed to 2 × 3 m in the other stands of the chronosequence. The growth curves were similar for all the stands and no biotic or abiotic factors severely affected their growth.

The oldest stand of the chronosequence was in a 50-ha plot. The downhill corner of this stand was growing in a clayey soil (from 37–40% clay in the A_1_ horizon to 42–45% down to a depth of 10 m). This corner was also studied 6 years after planting. The clayey soil was developed on basaltic material. Although weathered stones were found at a depth below 8 m (no stones were found down to a depth of 10 m in the chronosequence), fine roots were found between the stones down to a depth of 10 m. The stem biomass in the 6-year-old stand on clayey soil was 28% higher than in the 6-year-old stand on sandy soil (Campoe et al., 2012).

### Fine root sampling methodology

**Figure 1:**
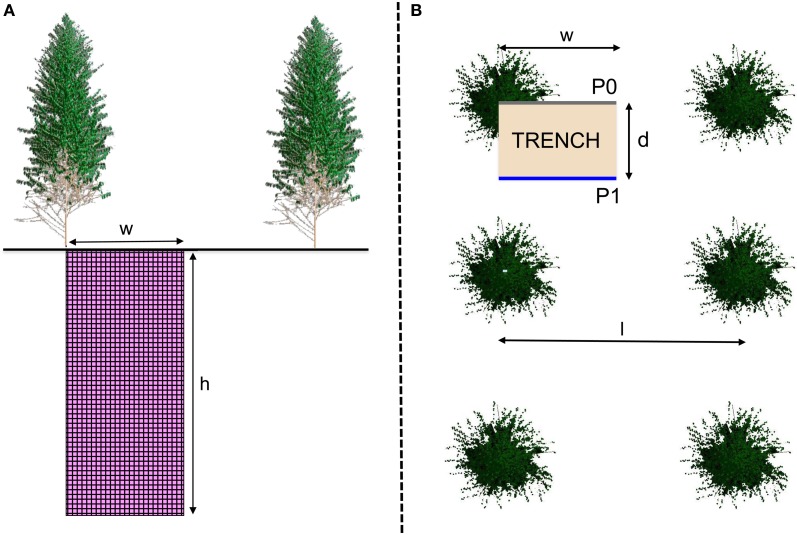
**Layout of soil profiles P0 (from the bottom of a trunk to the middle of the inter-row) and P1 (from midway between 2 trees in the planting row to the middle of the inter-row) in each stand showed in a side (A) and top view (B).** The distance between rows (*I*) was 300 cm and the distance between trees in the planting row (2*d*) was 200 cm, in the stands sampled 1, 2, and 3.5 years after planting. Spacing was slightly different in the 6-year-old stands (*I* = 380 cm and 2*d* = 160 cm) for a similar planting density. The maximum depth sampled (h) was 6 m in 1- and 2-year-old stands and 10 m in 3.5- and 6-year-old stands. The width of the soil profiles was I/2.

Three pits were dug in each stand close to three trees of mean basal area (no weeds or missing trees within a radius of 10 m). Root intersects were counted on two vertical trench walls at right-angles to the planting row in each pit: profile P0, from the bottom of the studied tree to the middle of the inter-row, and profile P1, from midway between two adjacent trees in the planting row to the middle of the inter-row (Figure 1). Three replicates of the P0 and P1 soil profiles (a total of 6 profiles observed for each stand age) were studied down to a depth of 1.5 m during the early growth phase (at 1 and 2 years after planting), and down to a depth of 3.0 m from mid-rotation onward (at 3.5 and 6 years after planting, on both sandy and clayey soils). Deep soil layers were sampled in 2 trench walls selected to represent extreme distances relative to trees (1 P0 and 1 P1 at each age) from 1.5 to 6 m deep at 1 and 2 years after planting, and from 3.0 to to 10.0 m deep at 3.5 and 6 years after planting. All vertical soil profiles were divided into 5 × 5 cm grid cells and roots were exposed using a small knife to remove surrounding soil. The number of intersections of roots with the vertical plane was counted in each grid cell of 25 cm^2^, distinguishing three sizes of diameter (fine roots less than 1 mm, medium-sized roots between 1 and 3 mm and coarse roots over 3 mm). Root classes were chosen as in previous studies carried out in Brazilian *Eucalyptus* plantations (e.g., da Silva et al., 2009; Laclau et al., 2013). Only living roots were counted on trench walls (as far as we could distinguish between living roots and dead roots from their color and flexibility). Root intersects were counted in 89,440 grid cells on 27 m^2^ of trench walls in 1- and 2-year-old stands, 48 m^2^ in 3.5-year-old stands, and 61 m^2^ in the 6-year-old stands on both sandy and clayey soils. Root growth is very fast in *Eucalyptus* plantations (Jourdan et al., 2008; Christina et al., 2011) and the roots counted in stands from 1 year after planting onwards belonged to several trees. The root characteristics were, therefore, representative of the stand and not only influenced by the nearest tree. The 3 pits studied at each age were more than 10 m from each other.

Fine root distribution was also studied by taking soil cores, on January 2012, down to a depth of 13 m at 2.1 years after replanting the oldest stand of the chronosequence. The methodology described by Christina et al. (2011) was used. Soils were sampled by drilling at a distance of 0.4, 0.9, and 1.5 m from 3 trees with the same basal area as the mean of the stand, along a diagonal between trees in adjacent rows. Only soil blocks from the central part of the auger were considered (the upper and lower parts were discarded), thus avoiding contamination from upper soil layers. Easily identifiable fine roots were separated by hand picking in the field. The soil samples were taken to the laboratory for thorough quantification of extremely fine roots. The root front was defined at each sampling position as the depth where the deepest root was observed.

### Water withdrawal from deep soil layers

The volumetric soil water content (SWC) was monitored at 30 min intervals in the oldest stand of the chronosequence established on sandy soil, before (March 2008–October 2009) and after (November 2009–October 2012) replanting. Forty-two CS616 probes (Campbell Scientific, Shepshed, England, UK) were installed: 5 probes in 5 pits at depths of 0.15, 0.50, and 1.00 m, 3 probes in 3 pits at depths of 2.00 and 3.00 m and 3 probes at different distances from trees in the same pit at depths of 4, 5, 6, 7, 8, 9, and 10 m. The pits were dug manually and the CS616 probes were buried horizontally in an undisturbed area from the vertical wall of each trench. The trenches were then back filled, keeping the soil horizons in their original positions. The probes were calibrated using gravimetric SWC and bulk density measurements.

The first occurrence of water withdrawal from deep soil layers after planting crops or trees has been used as an indicator of the root front displacement (Calder et al., 1997; Dardanelli et al., 1997; Battie-Laclau and Laclau, 2009). It was estimated that the age of the stand when the root front reached the soil moisture probes was shown by the first sharp decline in SWC. However, disruption of the supply of gravitational water to a given depth (resulting from water withdrawal by trees in the upper soil layers) could lead to a decrease in SWC, even though the roots may not have yet reached this depth. Therefore, only the depths where the SWC dropped to the lowest values observed before harvesting were taken into account in our study. It was considered that the root front reached the soil moisture probes at a given depth when an initial decrease in SWC due to the interruption of drainage from upper soil layers was followed by a sudden change in the slope of the SWC curve. In addition, the tree height was measured every 3 months in 4 plots (336 trees measured within a radius of 200 m from the soil moisture probes) to compare the vertical growth above- and belowground. The tree height was linearly interpolated to estimate the mean stand height each month throughout the study period.

### Data analyses

The numbers of intersects of fine and medium-sized roots per area of 25 cm^2^ of soil are presented as fine root density (FRD) and medium-sized root density (MRD). Coarse roots were not taken into account because they were only found close to the stump. The model proposed by Bouillet et al. (2002) was used to predict root intersects in trench walls throughout the development of eucalypt plantations:

(1) $${\text{FRD}}_{z}=a_{0}-a_{1}\times D_{z}+b\times \text{exp}(-c\times D_{z})+\varepsilon_{z},$$

where FRD_*z*_ is the mean FRD at depth *z*, *D*_*z*_ is depth *z*, (*a*_0_ + *b*) is the FRD at *D*_*z*_ = 0, (*a*_0_ − *a*_1_*D*_*z*_) tends to 0 when *D*_*z*_ increases, *c* controls the shape of the curve and ε is the residual error. As the relationship between the MRD and the soil depth was weak for most stand ages, only the means and standard deviations of MRD in each soil layer are shown.

Local and global fits of FRD models were compared between stand ages, soil profiles, and soil types. Models 1 (local models for each situation) and model 2 (global model for the whole data set) were fitted using SAS PROC NLIN. Differences in local and global models were evaluated using *F*-tests calculated on the residuals. This test is based on the error sum of squares (SSE) and the total number of parameters involved in the models. It compares *F*_obs_ and *F*_tab_ calculated as:

(2) $$F_{\text{obs}}=\frac{\left({\text{SSE}}_{2}-{\text{SSE}}_{1})/(p_{1}-p_{2}\right)}{{\text{SSE}}_{1}/(n-p_{1})},$$

where *p*_1_ is the number of parameters for the local model, *p*_2_ is the number of parameters for the global model (*p*_2_ < *p*_1_), SSE_1_ is the error sum of squares for the local model, SSE_2_ is the error sum of squares for the global model and *n* is the number of measurements. *F*_tab_ is the theoretical value given in Fischer's table: *F*_tab_ = *F*_(*p*1 − *p*2, *n* − *p*1)_. If *F*_obs_ > *F*_tab_ then the local model described the data set better than the global model and the factor studied had a significant effect (Brown and Rothery, 1993). All differences were considered significant at a 5% threshold.

The spatial distribution of roots was analyzed using classical univariate geostatistical methods including semivariogram analysis and interpolation (kriging) to describe spatial patterns (Isaaks and Srivastava, 1989). Semivariogram analyses were performed for omni-directional semivariograms. However, as the vertical gradient might have an effect on the spatial distribution, anisotropy was also studied by calculating directional semivariograms for the horizontal (X) and the vertical (Z) axes. Standardized semivariograms (standard semivariogram divided by the experimental sample variance for all spatial locations) were also calculated to compare results from different datasets. As the horizontal dimension was smaller (1.5 m) than the vertical dimension (6–10 m) in the soil profiles studied, spatial analyses were performed for 1.5 × 2.0 m areas in the top (0–2 m) layer, the middle (2–4 m) layer, and the bottom (4–6 m) layer of each soil profile. All geostatistical analyses were run using GS+ (Gamma Design Software, 2004). Semivariograms were modeled by fitting the parameters using the least-squares method (autofit facility of GS+). For each analysis, an average ratio of anisotropy (*R*) was calculated:

(3) $$R=\frac{1}{p}\sum_{i=1}^{i=n}\frac{\gamma_{X}(h_{i})}{\gamma_{Z}(h_{i})}$$

where *p* was the number of experimental semivariograms values calculated using an active lag distance set to 1 m and a lag class interval of 0.05 m (*p* = 19), *h*_*i*_ was the separation distance used to calculate the semivariograms, and γ_*X*_ and γ_*Z*_ were the directional semivariograms for the *X* and *Z* directions. *R*-values close to 1 indicated an isotropic spatial structure.

## Results

### Root front displacement

**Figure 2:**
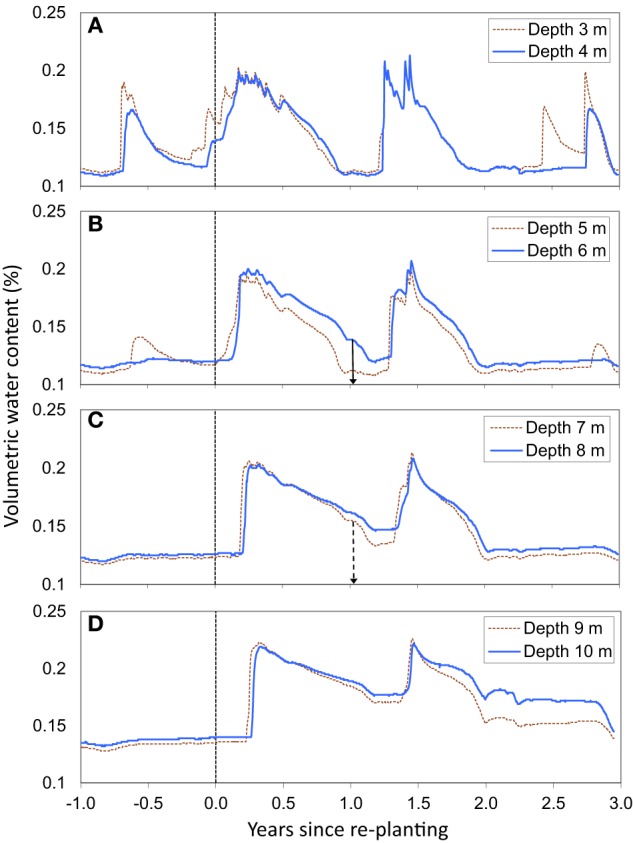
**Time series of volumetric soil water content at depths of 3 and 4 m (A), 5 and 6 m (B), 7 and 8 m (C), and 9 and 10 m (D).** Vertical arrows at depths of 6 and 7 m indicate the approximate age of the stand when water withdrawal at the root front led to a sharp decline in soil water content after stabilization resulting from a disruption of drainage from upper soil layers. The timecourse of soil water content at the other depths (3, 4, 5, 8, 9, and 10 m) did not make it possible to estimate the stand age at the arrival of the root front. Trees were harvested ~1 month before re-planting.

**Figure 3:**
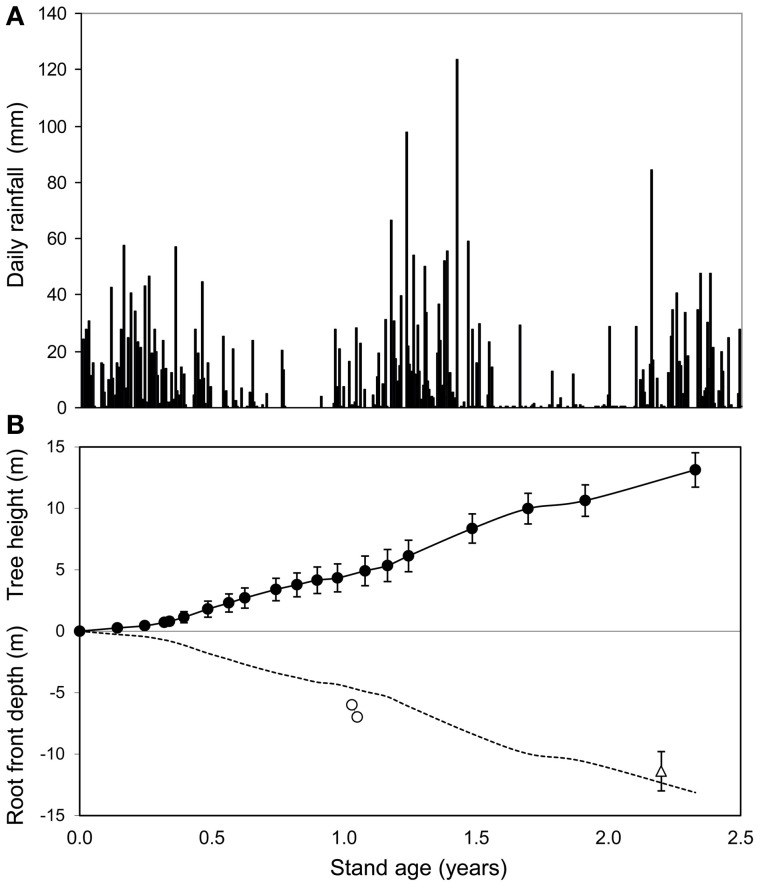
**Time series of daily rainfall (A) and mean stand height (filled circles) as well as root front depth (empty circles) estimated from soil water content monitoring and soil coring at 2.1 years (empty triangles) after replanting the oldest stand of the chronosequence (B).** Standard deviations of tree height (*n* = 336) and root front depth at age 2.1 years (*n* = 3) are shown. The dotted line shows the symmetrical belowground of mean tree height.

The SWC time series showed a fast displacement of the root front in deep soil layers (Figure 2). Gravitational drainage at the end of the first rainy season after planting led to a slow decline in SWC, at all depths between 3 and 10 m. There was a sharp acceleration in the decrease in SWC during the first dry season, reflecting the uptake of substantial amounts of water by tree roots (Figure 2). The SWC time series showed a displacement of the root front down to a depth of 7 m within 1.1 years after planting, which indicated a mean root growth rate downwards of approximately 1.8 cm day^−1^. The SWC down to a depth of 6–7 m at the end of the first dry period after planting (at about 1 year of age) was similar to values at the end of the dry season before clear cutting (Figures 2B, 3A). This pattern indicated that all the available water stored down to a depth of 6–7 m after clear-cutting the previous stand was already taken up 1 year after re-planting. The soil down to more than 10 m was replenished during the second rainy season after planting but gravitational water did not reach more than 5 m down during the third year after planting (Figure 2). Soil cores showed that the deepest roots reached a depth of 11.4 ± 1.6 m at 2.1 years after planting (Figure 3B). SWC monitoring at the depths of 6, and 7 m as well as soil coring at 2.1 years of age suggested a roughly symmetrical vertical extension of trees aboveground and belowground over the early growth of this *E. grandis* stand.

### Dynamics of soil exploration by roots

**Figure 4:**
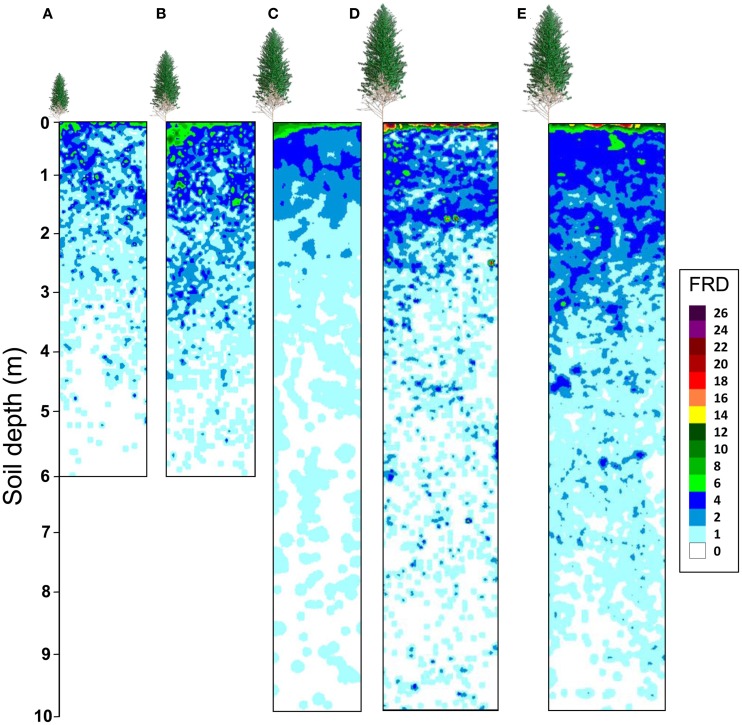
**Kriged maps of roots less than 1 mm in diameter (FRD, number of root intersects counted in a 25 cm^2^ area of trench wall) on the P0 soil profile.** Pits were at right angles to planting rows in a chronosequence of *Eucalyptus grandis* plantations down to a depth of 6 m at 1 and 2 years after planting (**A** and **B**, respectively) and down to a depth of 10 m in 3.5-year old **(C)** and 6-year-old **(D)** stands on a sandy soil (20% clay). Fine root densities down to a depth of 10 m in a 6-year-old stand on a clayey soil were also studied **(E)**. Eucalypts indicate the position of the nearest tree (not at the scale of soil profiles).

Maps of fine root densities confirmed fast exploration of deep soil layers throughout the development of *E. grandis* plantations with some fine roots observed at a depth of 6 m after only 12 months of growth (Figure 4). 1 and 2 years after planting, local models predicting the FRD for each soil profile (P0 and P1) were significantly different to global models based on both soil profiles, with a higher FRD in the soil profile at the bottom of a tree (P0) than in the profile further from the trees (P1) (Tables 2, 3). However, local and global models of FRD were not significantly different for the P0 and P1 profiles at 3.5 and 6 years after planting. This pattern suggested that soil exploration by roots was not greatly influenced by the distance from the nearest tree during the second half of the rotation cycle. Highly significant differences between local models predicting FRD at each stand age and a global model including all the ages showed a strong effect of stand development on the distribution of fine roots down to a depth of 10 m (Table 2). There was great spatial variability in FRD and MRD in deep soil layers. The coefficients of variation of FRD and MRD in 2 m thick soil layers below a depth of 2 m were >100 and >600%, respectively, for all stand ages and soil profiles (Table 3). Large changes in FRD distribution depending on tree age showed that the FRD tended to increase in the 0–5 cm soil layer at the end of the rotation cycle (Figure 4 and Table 3). The soil texture also had a significant effect on fine root distribution at 6 years of age (Table 2). The mean FRD down to a depth of 10 m was 40% higher in the clayey soil than in sandy soil (Figure 4). The MRD distribution was similar to the FRD distribution but the values were much lower and the variability was higher (Table 3).

**Table 2:** **Comparison of local and global models predicting fine root intersect densities (number of fine root intersects per 25 cm^2^ grid cell) across soil profiles, stand ages, and soil types**.

**Soil profile**	**Stand age (yr)**	**Soil type**	**Local models**	**R^2^**	**RMSE**	**Global models**	**R^2^**	**RMSE**	**F_obs_**
P0	1	20% clay	FRD = 1.4677 − 0.2960D + 6.7027exp(−19.0512D)	0.91	0.23	FRD = 1.3307 − 0.2587D + 5.3375exp(−19.3729D)	0.83	0.28	15.4^****^
P1			FRD = 1.1931 − 0.2213D + 3.9528exp(−19.7684D)	0.78	0.26				
P0	2	20% clay	FRD = 1.9000 − 0.3504D + 3.7492exp(−4.0025D)	0.83	0.24	FRD = 1.6917 − 0.3118D + 3.6078exp(−3.4614D)	0.94	0.25	9.4^****^
P1			FRD = 1.4520 − 0.9407D − 0.1275exp(−0.6502D)	0.95	0.22				
P0	3.5	20% clay	FRD = 2.7974exp(−0.7111D)	0.90	0.21	FRD = 2.7168exp(−0.7178D)	0.88	0.22	2.7 NS
P1			FRD = 2.6385exp(−0.7262D)	0.87	0.22				
P0	6	20% clay	FRD = 1.4419 − 0.1743D + 18.1805exp(−14.7543D)	0.92	0.36	FRD = 1.4221 − 0.1726D + 17.1942exp(−13.1137D)	0.92	0.36	2.2 NS
P1			FRD = 1.4023 − 0.1709D + 16.4777exp(−11.8440D)	0.93	0.35				
All	1	20% clay	FRD = 1.3307 − 0.2587D + 5.3375exp(−19.3729D)	0.83	0.17	FRD = 1.2318 − 0.1649D + 6.9213exp(−9.0045D)	0.69	0.54	330.0^****^
	2		FRD = 1.6917 − 0.3118D + 3.6078exp(−3.4614D)	0.94	0.25				
	3.5		FRD = 2.7168exp(−0.7178D)	0.88	0.22				
	6		FRD = 1.4221 − 0.1726D + 17.1942exp(−13.1137D)	0.92	0.36				
All	6	40% clay	FRD = 1.8160 − 0.2120D + 11.4810exp(−8.0817D)	0.91	0.38	FRD = 1.6472 − 0.1964D + 14.7334exp(−11.3966D)	0.90	0.40	39.9^****^
		20% clay	FRD = 1.4221 − 0.1726D + 17.1942exp(−13.1137D)	0.92	0.36				

*F-tests compare local models with a global model*.

Only parameters significantly different from 0 (P < 0.05) are shown. D is soil depth in meters. NS, ^*^, ^**^, ^***^ and

**** *indicate non-significant differences at P < 0.05 and significant differences at the thresholds of 0.05, 0.01, 0.001, and 0.0001, respectively*.

**Table 3:** **Mean, standard deviation (Std), and coefficient of variation (CV expressed in %) of fine and medium-sized root intersects counted in 25 cm^2^ grid cells (number of roots per 25 cm^2^) on the P0 (close to the stump) and the P1 (at mid distance between two trees in the planting row) soil profiles**.

**Age(yrs)**	**Soil layer (m)**	**Fine roots (diameter < 1 mm)**						**Medium-sized roots (diameter 1–3 mm)**					
		**P0**			**P1**			**P0**			**P1**		
		**Mean**	**Std**	**C.V.**	**Mean**	**Std**	**C.V.**	**Mean**	**Std**	**C.V.**	**Mean**	**Std**	**C.V.**
1	0.0–0.1	4.38	3.11	71	2.91	1.79	61	0.08	0.27	345	0.12	0.33	269
	0.1–0.3	1.68	1.44	85	1.11	1.20	108	0.03	0.16	625	0.02	0.15	664
	0.3–0.5	1.58	1.45	92	0.66	0.97	145	0.03	0.16	592	0.04	0.21	513
	0.5–1.0	1.48	1.37	92	0.91	1.03	113	0.04	0.20	526	0.03	0.16	618
	1.0–2.0	1.19	1.12	95	1.12	1.12	101	0.03	0.18	576	0.03	0.17	586
	2.0–4.0	0.38	0.67	176	0.46	0.84	183	0.02	0.16	747	0.02	0.15	684
	4.0–6.0	0.08	0.34	425	0.07	0.28	402	0.00	0.06	1732	0.00	0.05	1998
2	0.0–0.1	4.88	2.11	43	4.59	2.30	50	0.37	0.68	184	0.51	0.74	145
	0.1–0.3	3.72	2.02	54	3.37	1.95	58	0.14	0.36	249	0.18	0.46	263
	0.3–0.5	2.30	1.71	74	2.12	1.45	69	0.10	0.30	296	0.08	0.30	355
	0.5–1.0	1.89	1.42	75	1.75	1.49	85	0.07	0.26	398	0.07	0.25	378
	1.0–2.0	1.56	1.52	97	1.34	1.39	103	0.05	0.23	435	0.05	0.24	457
	2.0–4.0	0.76	0.86	113	0.57	0.78	136	0.00	0.06	1732	0.01	0.07	1411
	4.0–6.0	0.18	0.43	241	0.13	0.35	272	0.00	0.03	3478	0.00	0.00	
3.5	0.0–0.1	3.69	3.49	94	3.89	4.40	113	0.12	0.40	342	0.12	0.35	304
	0.1–0.3	2.05	1.97	96	1.67	1.21	73	0.03	0.18	650	0.06	0.23	413
	0.3–0.5	1.53	1.05	69	1.55	1.13	73	0.04	0.20	480	0.03	0.18	539
	0.5–1.0	1.75	1.08	62	1.70	1.13	67	0.05	0.24	435	0.04	0.21	483
	1.0–2.0	1.06	0.91	86	1.00	0.95	95	0.04	0.20	511	0.03	0.17	577
	2.0–4.0	0.31	0.62	202	0.32	0.56	175	0.01	0.10	956	0.01	0.12	947
	4.0–6.0	0.08	0.29	347	0.13	0.36	284	0.00	0.06	1545	0.00	0.03	3478
	6.0–8.0	0.07	0.28	402	0.09	0.30	343	0.00	0.04	2444	0.00	0.04	2444
	8.0–10.0	0.03	0.21	641	0.05	0.23	447	0.00	0.00		0.00	0.06	1545
6	0.0–0.1	10.34	5.62	54	11.24	6.20	55	0.55	0.97	178	0.50	0.95	191
	0.1–0.3	2.75	2.69	98	2.83	2.39	84	0.09	0.43	506	0.11	0.50	452
	0.3–0.5	1.63	1.27	78	1.82	1.62	89	0.07	0.27	410	0.08	0.78	954
	0.5–1.0	1.97	1.35	68	1.83	1.17	64	0.07	0.27	408	0.07	0.32	484
	1.0–2.0	1.62	1.23	76	1.57	1.09	69	0.05	0.23	449	0.05	0.24	479
	2.0–4.0	0.73	1.01	138	0.73	0.89	122	0.02	0.14	774	0.02	0.15	733
	4.0–6.0	0.28	0.60	211	0.26	0.61	233	0.01	0.10	1187	0.01	0.08	1202
	6.0–8.0	0.18	0.51	278	0.15	0.45	294	0.01	0.08	1261	0.01	0.09	1151
	8.0–10.0	0.11	0.37	336	0.09	0.34	383	0.00	0.06	1630	0.00	0.06	1630

*Root length densities (RLD, expressed in cm cm^−3^) can be estimated for fine roots using the formula fitted by Maurice et al. (2010) in the same stands: RLD = 1.89 LAI N_t_; where N_t_ is the root intersect density expressed as number of fine roots cm^−2^*.

**Figure 5:**
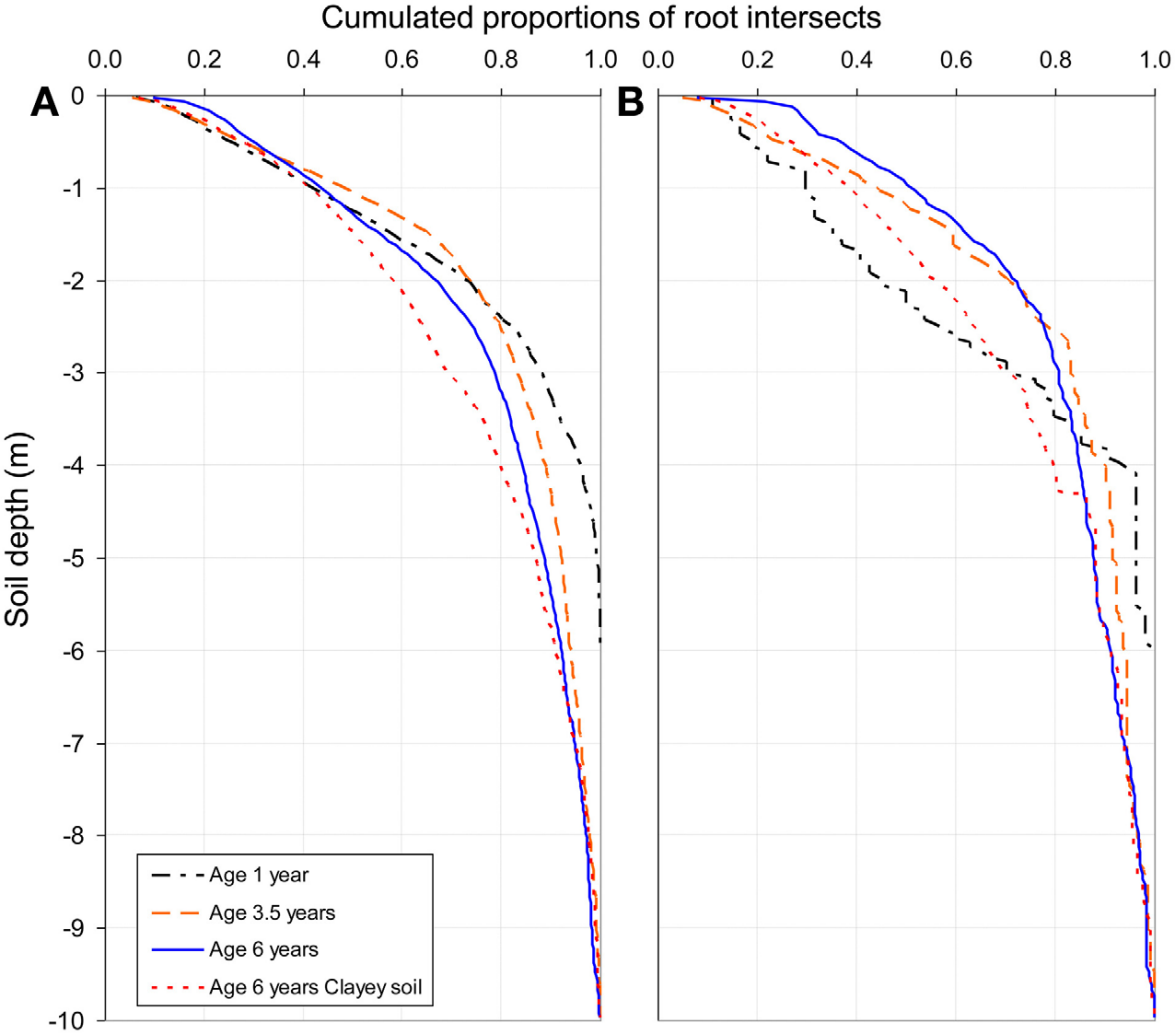
**Cumulative distribution of fine root intersects (diameter less than 1 mm, A) and medium-sized roots intersects (diameter 1–3 mm, B) down to a depth of 10 m, from observations on 2–6 soil profiles at each stand age.** Cumulative distribution of root intersects are not shown 2 years after planting because root intersects were only measured down to a depth of 6 m although soil coring showed the presence of roots lower down (fine root densities below 6 m were considered negligible at age 1 year).

Although the highest FRD was found in the top soil, less than 20% of the total fine root intersects down to a depth of 10 m were counted in the 0–50 cm soil layer in the 1-, 3.5-, and 6-year-old stands of the chronosequence (Figure 5A). Half of the total amounts of fine and medium-sized root intersects were found below a depth of 1.0–1.5 m in all the sampled stands (except for medium-sized roots at age 1 year). The proportion of fine roots below a depth of 4 m increased with stand age. They represented 5% of the total fine root intersects in the 1-year old stand, 10% in the 3.5-year old stand, 15% in the 6-year-old stand of the chronosequence and 20% in the 6-year-old stand on clayey soil. Medium-sized roots tended to accumulate in the upper soil layers throughout the stand development: about 30% of the cumulated medium-sized root intersects down to a depth of 10 m were found in the 0–1 m soil layer in the 1-year-old stand, 45% in the 3.5-year-old stand and 50% in the 6-year-old stand of the chronosequence (Figure 5B).

### Spatial exploration of soil by fine roots

**Figure 6:**
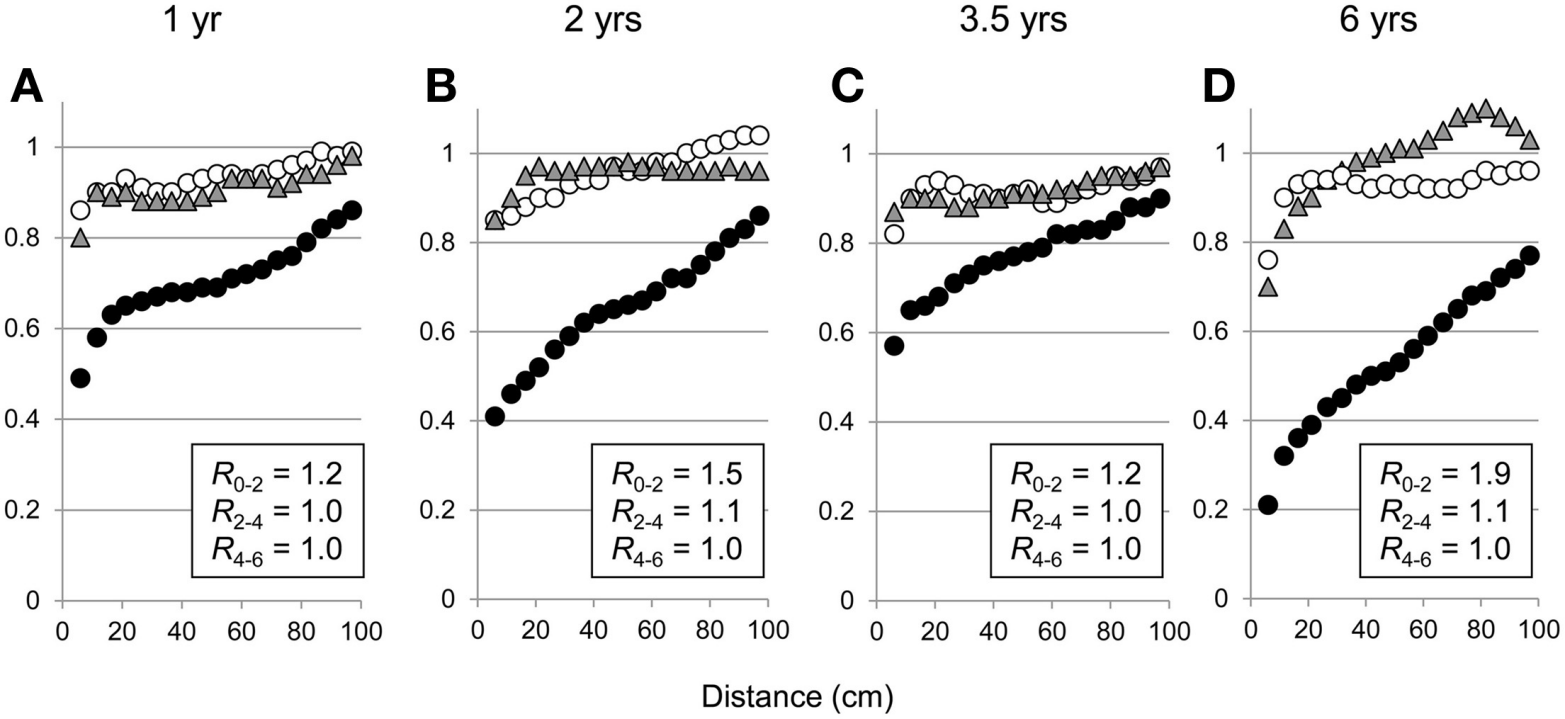
**Standardized variograms of fine root intersects in soil layers 0–2 m (filled circles), 2–4 m (filled gray triangles), and 4–6 m (empty circles) for the P0 soil profile in 1-, 2-, 3.5-, and 6-year-old stands on a sandy soil (A, B, C, D, respectively).** *R*_0−2_, *R*_2−4_, and *R*_4−6_ indicate the average ratios of anisotropy in soil layers 0–2, 2–4, and 4–6 m, respectively (see equation 3).

Standardized variograms showed a spatial dependence of fine roots in the upper layer (0–2 m) with an anisotropy increasing with stand age (Figure 6). Below a depth of 2 m, the spatial dependence of fine roots was weak, being limited to short distances (standardized variograms were horizontal for distances greater than 30 cm). Ratios of anisotropy close to 1.0 for all stand ages in the 2–4 and 4–6 m soil layers showed an isotropic exploration of deep soil layers by fine roots. Similar variograms at each age in the P0 and P1 soil profiles (data not shown) indicated that the spatial structure of fine root exploration was not strongly influenced by the distance from the nearest trees from 1 year after planting onwards. No spatial dependence was observed for medium-sized roots, whatever the stand age and the soil layer.

## Discussion

### Downward root growth rates

In accordance with the first hypothesis, the displacement of the root front in *E. grandis* plantations was fast in comparison to other plant species. Soil coring and SWC monitoring provided consistent estimates of root elongation rates (RER) downwards close to 2 cm day^−1^, which were similar to the mean height growth rates over the first two years after planting. A slight decrease in root front velocity with stand age in *E. grandis* plantations (as observed for height growth rates) might explain why the deepest roots were found at a depth of 9.5 m at 1.5 year after planting in Brazil (Christina et al., 2011) and at a depth of 28 m at 9 years of age in South Africa (Dye, 1996). High root front velocities have been reported for some herbaceous species growing in deep soils with no impediment to root growth. The SWC time series after an induced drought in a field experiment showed that the root front velocity peaked at 4.4 cm day^−1^ for sunflowers, 3.4 cm day^−1^ for soybeans, 3.0 cm day^−1^ for maize, and 2.3 cm day^−1^ for peanuts (Dardanelli et al., 1997). Sequential soil coring showed that the root front depth increased by 2.5 cm day^−1^ for sorghum and 4.1 cm day^−1^ for sunflowers, from 20 to 60 days after emergence (Stone et al., 2001). For sugarcane crops in Brazil, the mean root front velocity from 4 months after planting to harvesting was 1.9 cm day^−1^ (Battie-Laclau and Laclau, 2009). So far as we are aware, the root front velocity has never been measured for woody species growing in very deep tropical soils and the highest RER for tree species have been measured in pot experiments. The maximum values for rhizotron-grown *E. nitens* and *E. globulus* seedlings were about 2.5 cm day^−1^ (Misra, 1999). It has been shown for seedlings in rhizopods that lowering the water table encourages root elongation downwards for phreatophytic species (Stave et al., 2005; Canham, 2011). The maximum RER in rhizopods filled with a medium to coarse sand was 3.7 cm day^−1^ for *Banksia attenuata* seedlings and 1.8 cm day^−1^ for *Banksia littoralis* seedlings (Canham, 2011). RER reached 2.1 and 1.4 cm day^−1^ for seedlings of *Acacia tortilis* and *Faidherbia albida* in another experiment carried out in rhizopods (Stave et al., 2005). The mean root front velocity the first year after planting in our study was, therefore, close to the highest values reported for phreatophytic species in response to lowering the water table.

Although other studies have estimated the root front displacement from SWC time series (e.g., Calder et al., 1997; Dardanelli et al., 1997), it has only been possible to distinguish between the effects of a disruption of drainage from upper soil layers and water uptake by tree roots at depths of 6 and 7 m. The fact that SWC down to a depth of 6–7 m at ~1 year after planting was similar to the values at the end of the dry season before clear cutting could only be explained by substantial water withdrawal. The estimation of the root front depths from several approaches in our study (SWC time series at depths of 6 and 7 m, fine root distributions on trench walls 1 and 2 years after planting, and soil coring at age 2.2 years) were consistent and confirmed a synchrony between the vertical extension of shoots and roots already shown by Christina et al. (2011) in *E. grandis* plantations. The drop in SWC shown in the time series for depths of 8, 9, and 10 m, that was thought to have resulted only from the disruption of drainage from the upper soil layers, might also be caused by water withdrawal by tree roots (Figure 2). The water table was at a depth of 14 m from 1 to 1.3 years after planting (data not shown) and capillary rises in the soil (70% sand) could not account for the dynamics of SWC observed (Fan and Miguez-Macho, 2010). Further studies based on successive soil coring are needed to assess whether root front velocity in very deep soil layers increases during dry periods. In addition, stand evapotranspiration measured accurately by eddy-covariance at this study site could be used with a modeling approach to estimate the amounts of water stored in deep soil layers that are taken up throughout the rotation cycle.

High RER in deep soil layers in this study may be explained by a combination of favorable factors, including high water requirements in Brazilian *Eucalyptus* plantations (Cabral et al., 2010) for a GPP of about 4000 g C m^−2^ yr^−1^ at the study site (Campoe et al., 2012), favorable soil temperature and SWC for root growth in deep soil layers (Iijima et al., 1998; Thongo M'bou et al., 2008) and the lack of physical or chemical limitations on root growth in the soil. Recent studies in *Arabidopsis* plants showed that water potential gradients and/or moisture sensors are likely to trigger ABA and cytokinin signaling to modulate hydrotropism gene networks (Cassab et al., 2013). Stimulating the genes involved in root hydrotropism in response to the development of a gradient of soil water potential may account for high RER of *Eucalyptus* trees planted on land previously used for agriculture in Australia. A strong relationship was found between the mean tree height and lateral extent of roots of four commonly planted tree species (*E. globulus, Pinus radiata, Pinus pinaster*, and *E. kochii*) at 12 sites in Australian agro-forests. In particular, fine roots were found in the top soil up to a distance of 37 m from 15 m tall *Eucalyptus globulus* trees 6 years after planting (Sudmeyer et al., 2004). Horizontal RER in Australian agro-forests were, therefore, close to the values estimated for vertical RER in our study. Contrary to the pattern observed in Australian agro-forests, excavation of *E. grandis* superficial roots at our study site showed that the root lateral extension was less than 7 m, from 1 year after planting to harvesting (Christina et al., 2011). High inter-tree competition for water resources in the top soil in monospecific eucalypt plantations while large amounts of water are stored in deep soil layers after clear-cutting lead to the development of a vertical SWC gradient. It would be worthwhile studying the role of root hydrotropism in explaining high RER along gradients of soil water potential, horizontally in agro-forests and vertically in monospecific plantations.

### Spatial exploration of considerable soil volumes

In agreement with the second hypothesis, most of the soil volume was explored by fine roots in the upper 3 meters from 1 year after planting onwards. Counting root intersects on 3 faces of more than 1000 soil cubes (1 dm^3^ in volume) in the same *Eucalyptus* stands as used for this study, Maurice et al. (2010) showed that soil space occupation by fine roots was isotropic below a depth of 60 cm, while both isotropy and anisotropy could be found in the upper soil layers depending on stand age. This study indicated that fine root length densities were strongly correlated to root intersect densities on vertical soil profiles, even though the relationships depended on stand age and soil fertility. The relative FRDs between soil layers estimated from root intersect counts in our study were, therefore, probably similar for fine root length densities. Kriged maps showed that the development of anisotropic soil exploration in the upper layers led to fine root clustering, as shown by Bouillet et al. (2002) in *Eucalyptus* plantations and Schmid and Kazda (2005) in *Fagus sylvatica* and *Picea abies* forests. Severe soil hydrophibicity in the Congo led to root clumping in preferential drainage channels under *Eucalyptus* plantations, which helped to explain a rapid nutrient uptake from soil solutions (Laclau et al., 2001). In the present study, clustered fine roots in the top soil probably reflected a concentration of resources throughout stand growth. Whilst gravitational water reached depths of 10 m in the first 2 years after planting, it did not reach 6 m deep thereafter. Although fertilizers are applied to the top soil in *Eucalyptus* plantations, significant amounts of potassium and nitrate are leached to a depth below 1 m in sandy soils (Silva et al., 2013), and taken up by tree roots between depths of 1 and 3 m (Laclau et al., 2010). From 2 years after planting onwards, the biological cycle leads to an accumulation of nutrients in the top soil (Laclau et al., 2003) and water availability is low between the depths of 5 and 10 m (Figure 2). The spatial variation in FRD throughout the rotation in tropical *Eucalyptus* plantations is, therefore, well-suited to prevent water and nutrient losses by deep drainage. A functional specialization of fine roots, with a higher capacity to take up Sr^2+^ and Rb^+^ (analogs of Ca^2+^ and K^+^, respectively) in deep soil layers rather than in top soil layers, also helps to prevent nutrient losses in *E. grandis* stands (da Silva et al., 2011). The functional role of deep roots in these plantations has been confirmed by modeling approaches, which show that the predictions of production are greatly improved when water storage in very deep soil layers is taken into account (Mendham et al., 2011; Marsden et al., 2012).

*E. grandis* trees explored a considerable volume of soil with limited carbon cost. Fine roots below a depth of 4 m accounted for less than 20% of the total fine root intersects down to 10 m, for all stand ages. Despite a tendency toward fine root clumping in deep soil layers (spatial dependence less than 30 cm below a depth of 2 m), the SWC time series showed that low fine root densities had the capacity to withdraw large amounts of water. Fine root clumping in soil areas of preferential infiltration of gravitational water through the top soil, as well as the development of a superficial root mat in the forest floor (Laclau et al., 2004) may also help to prevent water and nutrient losses after canopy closure in tropical *Eucalyptus* plantations. The plasticity in soil exploration by fine roots throughout tree growth probably plays a major role in maximizing resource use in these fast-growing plantations.

To conclude, this study shows very fast soil exploration by fine roots down to a depth of 10 m in *E. grandis* plantations. All the water available for trees that was stored down to a depth of 6–7 m after clear cutting was withdrawn during the first year after planting. High FRD in the upper 3 m of soil and sparse clustered fine roots in very deep soil layers made it possible to prevent water loss by deep drainage after canopy closure. These results suggest that the functional role of deep roots has not been sufficiently taken into account by forest managers. The soil water holding capacity down to depths greater than 10 m is an important criterion to select the most suitable land for afforestation and to improve the predictions of biomass production by process-based models. Further studies of the anatomical, architectural and functional characteristics of fine roots along very deep soil profiles should be carried out to gain an insight into their potential impact on C, water and nutrient cycles in tropical regions.

### Conflict of interest statement

The authors declare that the research was conducted in the absence of any commercial or financial relationships that could be construed as a potential conflict of interest.
